# Transport-capacity thresholds for urban nutrition security under extreme food-supply disruptions: evidence from Shanghai

**DOI:** 10.3389/fnut.2026.1868446

**Published:** 2026-07-14

**Authors:** Hao Zhang, Qijun Jiang

**Affiliations:** College of Economics and Management, Shanghai Ocean University, Shanghai, China

**Keywords:** emergency food supply, healthy diet, megacity resilience, nutrition security, Shanghai, transport-capacity threshold, urban food systems

## Abstract

Urban nutrition security is increasingly vulnerable to extreme weather, public health emergencies, and interruptions to interregional food logistics, especially in import-dependent megacities. Guided by the Greater Food Approach, a Chinese policy framework that extends food security from grain sufficiency to diversified food sources, nutrition, and resilience, this study develops a scenario-based, nutrition-constrained assessment of Shanghai’s emergency food-security capacity in 2023, focusing on a minimum energy- and macronutrient-balanced emergency standard rather than comprehensive nutritional adequacy. We combine a baseline net-inflow model, a static minimum transport-capacity ratio model, a nutrition-constrained emergency ration optimization model, and a dynamic response-threshold framework to identify food-category bottlenecks under observed consumption, healthy-diet targets, and a minimum energy- and macronutrient-balanced emergency standard. Shanghai’s food system is structurally dependent on extra-local inflows: edible vegetable oil, beef and mutton, poultry, eggs, milk, aquatic products, and fruit all show high external dependence, whereas vegetables retain stronger local buffering capacity. Under normal-consumption targets, the principal bottlenecks are meat subcategories, with the system requiring about 70% of normal external transport capacity to maintain observed supply. Under the healthy-diet target, bottlenecks shift to fruit and milk, and fruit reaches a model-derived planning reference value of 1.33, suggesting that guideline-consistent fruit supply would require transport and supply support beyond the current normal benchmark under the specified assumptions. Under the benchmark callable-reserve assumption, local production and estimated reserves are sufficient in the model to satisfy the minimum energy- and macronutrient-balanced emergency standard for 15 days, while the model-derived bottleneck transport reference values rise to 0.34, 0.62, and 0.84 for 21-, 30-, and 45-day disruptions, respectively. These findings suggest that, under explicit scenario assumptions, emergency food resilience in Shanghai and comparable import-dependent megacities may depend not only on reserve size, but also on bottleneck food-category configuration, nutrient-equivalent substitution, reserve-release accessibility, and the minimum availability of external logistics corridors.

## 1 Introduction

Ensuring access to nutritionally adequate food during extreme disruptions is a central challenge for megacity governance. In 2024, the General Office of the State Council issued the Opinions on Practicing the Greater Food Approach and Building a Diversified Food Supply System, marking a policy shift from a narrow focus on staple-grain sufficiency toward a broader food-security concept that integrates quantity, diet quality, nutrition, and resilience (1–3). This shift is consistent with the emerging nutrition-security literature, which emphasizes not only sufficient food availability but also diet quality, nutritional adequacy, and equitable access to health-promoting foods (4). This orientation is particularly relevant for megacities, where dense populations, diversified dietary demand, limited local production capacity, and long supply chains make nutrition security highly dependent on external food flows (5). Extreme rainfall, epidemics, regional logistics interruptions, and other compound shocks can rapidly create urban supply isolation, especially when mobility restrictions and transport bottlenecks disrupt food access even before aggregate food availability collapses (6, 7). Recent Chinese urban emergencies also show persistent vulnerabilities in last-mile delivery, interregional coordination, and multi-category dispatch (8). A key question is therefore how much external transport capacity must remain available for an import-dependent megacity to protect basic nutrition under extreme food-supply disruptions.

Existing research increasingly evaluates food security through nutrition rather than aggregate weight alone, reflecting a broader conceptual shift from food availability to food and nutrition security (9). Liang and Chen (10) traced calorie, fat, and protein supply–demand patterns in China through a nutritional conversion framework. Zhou et al. (11) estimated food self-sufficiency under nutritionally balanced dietary targets. Xiong and Wang (12) proposed a three-dimensional framework covering edible, trade, and nutritional food gaps, emphasizing the role of dietary restructuring and resource efficiency. However, most of this literature focuses on national or regional food systems. In megacities, supply–demand mismatches transmit more directly because of concentrated consumption, limited self-sufficiency, and stronger dependence on interregional logistics. Urban food systems are nested networks spanning global, national, regional, wholesale, retail, and community scales (13), and network-based approaches have revealed source diversity, multicentric organization, and nodal vulnerabilities in large urban regions (14). Regional coordination and emergency-support hubs are therefore central to sustaining urban food security under spatial concentration (15). City-region food system studies further show that urban–rural coordination, infrastructure, and multi-level governance are key to food-system resilience under shocks (16). Yet the nutrition-security implications of transport-capacity loss remain underexplored.

Emergency-management and emergency-logistics studies have examined material distribution, reserve-network optimization, and allocation efficiency under disasters and public-health emergencies. Prior work has addressed supply–demand imbalance in emergency logistics (17), two-echelon delivery under uncertain demand (18), reserve-depot network optimization in urban agglomerations (19), emergency-food functions and standards (20, 21), and governance of grain reserves (22). These studies offer important insights into emergency distribution and reserve management, but fewer studies embed minimum nutritional requirements into transport-threshold identification. Studies of pandemic-related food supply chains show that disruptions can occur across production, processing, distribution, retail, and consumption stages (23). As a result, existing frameworks often identify whether food volumes can be supplied, while paying less attention to whether emergency portfolios remain nutritionally adequate when external logistics are constrained. International debates on healthy diets and sustainable food systems also emphasize that dietary adequacy should be assessed jointly with food-system capacity and resilience (24).

This study reframes megacity emergency food provision as a nutrition-security problem and uses Shanghai as the empirical case. We develop an integrated framework linking the Greater Food Approach, healthy-diet targets, a minimum energy- and macronutrient-balanced emergency standard, local production, reserve stocks, and external transport-capacity constraints. This framing follows the broader concept of food-system resilience, which concerns the capacity of food systems to provide sufficient, appropriate, and accessible food under shocks and disturbances (25). In this study, nutrition security is used as the broader conceptual frame, whereas the quantitative model operationalizes a narrower minimum emergency standard based on energy sufficiency and macronutrient balance. The model therefore evaluates whether a minimum energy- and macronutrient-balanced emergency ration can remain feasible under transport constraints, rather than whether comprehensive nutritional adequacy can be achieved. Micronutrients, dietary diversity, food acceptability, cultural suitability, and vulnerable-group-specific needs are important dimensions of nutrition security, but they remain beyond the scope of the present optimization framework. First, we calculate baseline net inflows and external-dependence structures under normal-consumption and healthy-diet targets. Second, we formulate a nutrition-constrained emergency ration optimization model to identify feasible food-category combinations when transport capacity is severely limited. Third, we incorporate reserve release under different disruption durations to estimate static and dynamic transport-capacity thresholds and to develop a scenario-based planning framework. The contribution is threefold: the study operationalizes nutrient-equivalent substitution in an urban emergency food-security model; quantifies transport-capacity reference thresholds required to sustain a minimum energy- and macronutrient-balanced emergency standard in Shanghai; and provides a scenario-based planning framework that links disruption duration, bottleneck food categories, and emergency food-security response.

## 2 Materials and methods

### 2.1 Analytical framework

From the perspective of the Greater Food Approach, urban food-security assessment should not be limited to static supply–demand balance under normal conditions. It should also evaluate whether alternative food portfolios can maintain a minimum energy- and macronutrient-balanced emergency standard when supply chains are disrupted. The present framework therefore covers three targets: observed normal consumption, the healthy-diet target recommended by the Dietary Guidelines for Chinese Residents (26), and the minimum energy- and macronutrient-balanced emergency standard under emergency conditions. Baseline net inflows are used to identify structural external dependence. Static minimum transport-capacity ratios are estimated after accounting for reserve stocks. Sustained-disruption scenarios then combine reserve-release constraints with a nutrition-constrained linear programming model to identify the minimum transport capacity needed to preserve the minimum energy- and macronutrient-balanced emergency standard over 15-, 21-, 30-, and 45-day horizons. Finally, static thresholds, dynamic rationing results, and duration-specific reference points are combined into a scenario-based emergency food-security planning framework.

### 2.2 Baseline net inflows and static minimum transport-capacity ratios

To characterize the structural dependence of a megacity on extra-local food-supply chains and provide a baseline for transport-stress tests, this study first constructs a baseline net-inflow model (27). Let I denote the set of major food categories, including grains, edible vegetable oil, meats, eggs, milk, aquatic products, vegetables, and fruit. Let G denote the set of food-security targets, specifically the observed normal-consumption target and the healthy-diet target based on the Dietary Guidelines for Chinese Residents.

#### 2.2.1 Baseline net inflows

The baseline net inflow represents the minimum amount of each food category that must be brought into the city from outside its administrative boundary in order to maintain a given consumption target under normal logistics and market conditions. This indicator not only reflects the city’s structural dependence on external food provisioning, but also provides the fundamental variable for evaluating disruption risks under extreme scenarios. For any food category i under target g , the per capita baseline net inflow is defined as


$$M_{i}^{g}=\max(0,d_{i}^{g}-p_{i})$$

where $d_{i}^{g}$ denotes per capita daily demand for food category i under target g , and $p_{i}$ denotes per capita daily effective local supply after deducting inedible portions and normal production-circulation losses from reported production. When local supply is insufficient to meet demand, $M_{i}^{g}$ captures the rigid inflow requirement; otherwise, it is set to zero. External dependence can be further defined as


$$D_{i}^{g}=\frac{M_{i}^{g}}{d_{i}^{g}}$$

which measures the structural dependence of each food category on external supply chains (28).

#### 2.2.2 Static minimum external transport-capacity ratios under the total-stock assumption

In most public emergencies and natural disasters, urban food-security systems depend on existing logistics infrastructures, including external corridors, wholesale markets, cold-chain nodes, and community distribution networks (29). Building on the baseline net inflow, this study introduces reserve stocks to estimate the minimum share of normal external transport capacity that must remain available for each food category under different nutrition-security targets. Let $R_{i}$ denote the total reserve stock of category i . The category-level static minimum transport-capacity ratio is defined as


$$K_{i}^{g}=\max\{0,\frac{d_{i}^{g}-p_{i}-R_{i}}{M_{i}^{g}}\}$$

If local supply and reserves are already sufficient to cover target demand, external transport dependence for that category is recorded as zero. At the system level, the static minimum external transport-capacity ratio is determined by the most restrictive bottleneck category:


$$K^{g*}=\max_{i\in I}K_{i}^{g}$$

This indicator captures the minimum proportion of normal external transport capacity that must be retained, under the total-stock assumption, to sustain a given nutrition-security target. A higher value indicates stronger dependence on external logistics corridors and weaker buffering capacity against disruption.

### 2.3 Nutrition-constrained emergency ration optimization model

Linear programming has been widely used in nutrition research to construct food portfolios that satisfy nutrient constraints under alternative objective functions (30). When external logistics corridors remain constrained for a sustained period, the objective of urban food security shifts from maintaining the observed consumption structure to protecting a minimum energy- and macronutrient-balanced emergency standard. To represent substitution across food categories and dependence on external logistics under sustained disruption, this study develops a deterministic linear programming model for emergency ration optimization (31, 32). The model sets a minimum energy requirement of 2,100 kcal person-1 day-1 and imposes interval constraints on the energy shares of protein, fat, and carbohydrates. It therefore represents a minimum macronutrient-balanced emergency standard, rather than a comprehensive nutrition-security assessment that includes micronutrients, dietary diversity, acceptability, or vulnerable-group-specific requirements. All linear programming models and threshold calculations were implemented and solved in Stata 18.

To evaluate the robustness of the dynamic transport-capacity thresholds, we further conducted sensitivity analyses on three influential assumptions: reserve duration, fruit and vegetable inflow allowance, and the minimum energy requirement. Reserve duration was varied from a low-reserve case of 10 days for grains and edible oil and 2 days for perishable foods to a high-reserve case of 20 days for grains and edible oil and 4 days for perishable foods. The benchmark exclusion of external fruit and vegetable inflows was relaxed by allowing 10, 20, and 30% of normal net inflows. The minimum energy requirement was varied from 2,000 to 2,200 kcal person-1 day-1 while keeping the same macronutrient energy-share ranges.

Let $x_{i}$ denote the per capita daily ration allocated to category i , $y_{i}$ the per capita daily external inflow of category i , $R_{i}$ total reserves, and H the duration of the sustained disruption. The maximum daily reserve that can be mobilized for category i under disruption duration H is given by:


$$r_{i}(H)=\frac{R_{i}}{H}$$

This formulation represents an idealized average daily callable reserve and is used as a benchmark for comparing disruption durations. It should not be interpreted as evidence that all reserve stocks can be immediately transformed into usable daily supply. In practice, reserve mobilization may be constrained by storage quality, spoilage, cold-chain capacity, distribution bottlenecks, uneven spatial accessibility, and institutional coordination delays. The reserve term therefore represents potential callable capacity under benchmark assumptions rather than guaranteed deployable supply. This study considers four disruption-duration scenarios, namely H=15 , H=21 , H=30 , and H=45 , to capture variations in average daily reserve-release capacity across short-, medium-, and longer-duration disruptions. The supply constraints are specified as:


$$p_{i}\leq x_{i}\leq p_{i}+r_{i}(H)+y_{i},\;\;0\leq y_{i}\leq M_{i}^{0}$$

where $M_{i}^{0}$ denotes the baseline per capita daily net inflow under the normal-consumption target. Thus, reserves are treated as a callable upper bound rather than as a quantity that must be fully deployed on each day.

Let K denote the set of key nutrients and $A=(a_{\mathrm{ik}})$ the nutrient-density matrix for representative staple and non-staple foods, where $a_{\mathrm{ik}}$ denotes the amount of nutrient k contained in one unit of effective edible quantity of category i . Different food items are thereby projected into a common nutritional space. The nutrition constraints are written as:


$$\sum_{i\in I}a_{\mathrm{ik}}x_{i}\geq b_{k}^{\min},\;\forall k\in K_{\min}$$

$$\sum_{i\in I}a_{\mathrm{ik}}x_{i}\leq b_{k}^{\max},\;\forall k\in K_{\max}$$

where K_min and K_max denote the sets of lower- and upper-bound constraints, respectively, and b_k^min and b_k^max are the threshold levels of nutrient requirements after conversion into nutrient mass or energy-share terms. In the benchmark emergency-ration model, external inflows of vegetables and fruit are set to zero as a conservative lower-bound stress-test assumption. This assumption is motivated by the greater dependence of these categories on freshness, cold-chain coordination, short distribution cycles, and last-mile retail access during extreme disruptions. It does not imply that emergency logistics systems would fully exclude vegetables or fruit in practice. In actual emergency management, these categories may be partially prioritized, preserved, rerouted, or substituted through alternative procurement channels. The zero-inflow setting is therefore used only to examine a severe benchmark case in which limited transport capacity is prioritized for energy-dense and macronutrient-critical foods, while vegetables and fruit are supplied only through local production and releasable reserves. To evaluate the influence of this assumption, the sensitivity analysis allows partial vegetable and fruit inflows as a share of their baseline net inflows.

To reflect different emergency-management priorities, two optimization objectives are considered.

#### 2.3.1 Scenario 1: minimum total external inflow

This scenario identifies the theoretical lower bound of dependence on external physical supply while still meeting the minimum energy- and macronutrient-balanced emergency standard under nutrition constraints. The objective is:


$$\min\sum_{i\in I}y_{i}$$

#### 2.3.2 Scenario 2: minimum system bottleneck transport ratio

This scenario is designed to capture system bottlenecks when transport capabilities vary across food categories. By minimizing the occupancy ratio of the most stressed transport corridor, it identifies the minimum system bottleneck transport ratio. Introducing the auxiliary variable t , where t represents the maximum corridor-occupancy ratio at the system level, the model is specified as:


mint

$$y_{i}\leq tM_{i}^{0},\;\forall i\in I$$

In substance, this scenario searches across all feasible solutions that satisfy nutritional constraints for the minimum value of the most stressed transport corridor. The corridor-capacity constraint ensures that the occupancy ratio of any food category does not exceed t, so the optimal solution for t is the minimum system bottleneck transport ratio. To avoid multiple equivalent solutions and keep physical inflow volumes as low as possible once the bottleneck ratio is minimized, a two-stage optimization strategy is used: t is first minimized, and total external inflow is then minimized conditional on the optimal value of t .

### 2.4 Dynamic response-threshold identification and scenario-based planning rules

The thresholds derived in this section should be interpreted as scenario-based analytical constructs rather than empirically validated operational breakpoints. They are designed to compare relative stress levels under explicit assumptions, not to prescribe automatic administrative response triggers. Static supply–demand gaps capture the immediate pressure generated when local supply becomes insufficient and external inflows are disrupted. They do not reveal how long reserves can support the system, how much external transport capacity must be retained under sustained disruption, or when emergency targets should shift from observed consumption to the minimum energy- and macronutrient-balanced emergency standard. Building on the supply, reserve, and nutritional constraints defined above, this study introduces disruption duration and system bottlenecks to identify dynamic response thresholds for the minimum energy- and macronutrient-balanced emergency standard.

The dynamic response threshold $\theta_{H}$ is defined as the minimum possible occupancy ratio of the most stressed transport corridor under a sustained disruption of duration H :


$$\theta_{H}=\min\;t$$

subject to


$$y_{i}\leq tM_{i}^{0},\;\forall i\in I$$

and all nutritional, supply, and feasibility constraints defined above. The resulting $\theta_{H}$ indicates the minimum share of normal external transport capacity that must remain available to sustain the minimum energy- and macronutrient-balanced emergency standard when the disruption lasts for H days. A larger $\theta_{H}$ indicates stronger dependence on recovery of external logistics and greater vulnerability of key transport corridors.

Static and dynamic thresholds are then combined to build a graded scenario-based planning rule. Let $K^{C*}$ denote the system-level static minimum transport-capacity ratio under the normal-consumption target, $K^{H*}$ the corresponding ratio under the healthy-diet target, $\theta_{21}$ the dynamic response threshold under the 21-day disruption scenario, and κ the actually available share of external transport capacity. The scenario-based planning rule is defined as


$$\begin{array}{l} L(\kappa )=\\ \left\{ \begin{array}{ll} \text{Level}\;\mathrm{III}:\text{pressure}\;\mathrm{on}\;\text{normal food}-\text{security operations}, & K^{C*}\leq \kappa <K^{H*}\\ \text{Level}\;\mathrm{II}:\text{structural shortage warning}, & \theta_{21}\leq \kappa <K^{C*}\\ \text{Level}\;I:\text{crisis in minimum food}-\text{security standard}, & 0\leq \kappa <\theta_{21} \end{array}\right. \end{array}$$

In this framework, $K^{H*}$ primarily serves as a planning reference boundary for the healthy-diet target rather than as a rigid emergency trigger. By contrast, $\theta_{30}$ and $\theta_{45}$ are treated as additional reference points that reveal how dynamic transport-capacity boundaries shift upward under medium- and longer-duration disruptions. The resulting planning rules integrate normal-operation reference points, the minimum macronutrient-balanced emergency boundary, and disruption duration into a common analytical framework. They are intended to support scenario comparison and preparedness planning, rather than to define automatic emergency-response stages.

### 2.5 Sensitivity analysis

To examine the robustness of the scenario-based thresholds, three sets of one-way sensitivity analyses were conducted. First, reserve duration was varied to represent alternative assumptions about reserve availability: a low-reserve case with 10 days of coverage for grains and edible oil and 2 days for perishable foods, the baseline case with 15 days of coverage for grains and edible oil and 3 days for perishable foods, and a high-reserve case with 20 days of coverage for grains and edible oil and 4 days for perishable foods. Second, the benchmark zero-inflow assumption for vegetables and fruit was relaxed by allowing external inflows equal to 10, 20, and 30% of their baseline net inflows. Third, the minimum energy requirement was varied from 2,000 to 2,200 kcal person-1 day-1 while keeping the same macronutrient energy-share ranges. For each sensitivity scenario, the model recalculated the 21-day, 30-day, and 45-day dynamic bottleneck transport reference values and examined whether the ordering of the main thresholds remained stable.

### 2.6 Data sources

The study draws on five types of data: population characteristics, food consumption, agricultural supply, reserve stocks, and nutritional parameters. Data on Shanghai’s resident population and major agricultural outputs are taken from the Shanghai Statistical Yearbook and converted into per capita daily supply figures. To correct for the undercoverage of away-from-home consumption in official household statistics, this study follows the treatment used in China and Global Food Policy Report and uses the China Health and Nutrition Survey to estimate away-from-home consumption shares across major food categories for urban and rural residents from 1997 to 2011. These estimates are then combined with income elasticities of away-from-home food consumption derived by Zheng and Zhao (33) and Huang and Xie (34) to calibrate Shanghai’s 2023 food-consumption structure. Milk consumption is converted into raw-milk equivalent using the China Dairy Quality Report (2024) to ensure consistency between demand and supply statistics. For oil crops, rapeseed output is converted into rapeseed-oil equivalent using an extraction rate of 45% (35).

Reserve-stock estimation follows policy-based and standards-based assumptions rather than directly observed emergency deployment data. Reserves of finished grain and edible oil are calculated according to the minimum requirement of maintaining more than 15 days of market supply in 36 large and medium-sized cities as stipulated by China’s National Food and Strategic Reserves Administration. For perishable non-staple foods, including meat, eggs, milk, vegetables, and fruit, reserve levels are estimated with reference to the Guidelines for Disaster Relief Material Reserve issued in Shenzhen (36), assuming reserve coverage for 10% of the resident population for three days. These assumptions are combined with Shanghai’s population size and per capita food demand to estimate reserve quantities for each food category, which are then converted into per capita physical quantities. Because actual reserve data and emergency deployment records are not fully observable, these estimates should be interpreted as benchmark reserve scenarios rather than measured operational stocks. Static threshold identification uses total reserves directly as the system buffer, whereas sustained-disruption scenarios convert reserves into per capita daily callable quantities by dividing total reserves by disruption duration.

For nutritional parameters, recommended intake levels under the healthy-diet target are taken from the Dietary Guidelines for Chinese Residents (2022) and set at the midpoint of the recommended ranges. To account for inedible portions and normal losses, effective edible ratios are set at 90% for grains, 85% for meats, 89% for eggs, 54% for aquatic products, 82% for fruit, and 77% for vegetables (12). Under the minimum energy- and macronutrient-balanced emergency standard, the minimum energy requirement is set at 2,100 kcal person^−1^ day^−1^. The permissible energy-share ranges for protein, fat, and carbohydrates are determined with reference to the Dietary Reference Intakes for Chinese Residents and the relevant emergency nutrition standards of UNHCR (37, 38). Specifically, protein contributes 10–15% of total energy, fat 20–30%, and the residual energy is provided by carbohydrates. Energy and macronutrient data for each food category are obtained from the Chinese Nutrition Society food-composition database (Table 1).

**Table 1:** Energy and macronutrient contents of major food categories per 100 g of effective edible portion.

Food category	Energy (kJ)	Protein (g)	Fat (g)	Carbohydrate (g)	Data source
Grains	1276.00	12.86	4.46	55.18	Average of wheat, rice, maize, potatoes, and soybeans
Edible vegetable oil	3693.00	0.00	99.80	0.00	Average of rapeseed oil, peanut oil, and soybean oil
Eggs	599.00	13.30	8.80	2.80	Average egg values
Milk	227.00	3.00	3.20	3.40	Average cow’s milk values
Pork	1634.00	13.20	37.00	2.40	Average for pork, lean and fat combined
Poultry	698.00	19.30	9.40	1.30	Average chicken values
Beef	528.00	19.90	4.20	2.00	Average for beef, lean and fat combined
Mutton	845.00	19.00	14.10	0.00	Average for mutton, lean and fat combined
Aquatic products	361.00	14.95	1.68	2.63	Average of fish, shrimp, crab, and shellfish
Vegetables	129.50	2.03	0.28	5.65	Average of root, leafy, solanaceous, and cauliflower-type vegetables
Fruit	276.75	0.68	0.18	15.95	Average of grapes, oranges, bananas, and apples

## 3 Results

### 3.1 Food-consumption structure and external dependence

Table 2 shows that the supply–demand relationship for staple and non-staple foods in Shanghai in 2023 was strongly dominated by external inflows. Per capita daily grain consumption reached 462.11 g, whereas local daily supply was only 112.21 g. Baseline daily net inflows amounted to 349.90 g, yielding an external dependence ratio of 75.72%. Local supply of edible vegetable oil was almost negligible, while daily net inflows reached 57.22 g and external dependence rose to 99.75%, making edible oil the most externally dependent basic energy source in the system. External dependence also remained high for eggs, milk, aquatic products, and fruit, at 93.94, 87.34, 81.80, and 84.96%, respectively. Dependence was even more pronounced for meats. Per capita daily consumption was 132.09 g for pork, 87.47 g for poultry, and 66.19 g for beef and mutton combined. The corresponding baseline net inflows were 120.26 g, 85.88 g, and 65.92 g, yielding external dependence ratios of 91.04, 98.19, and 99.60%, respectively. This indicates that animal-source foods as a whole rely heavily on inflows from outside the municipality. By contrast, vegetables retained a much stronger local base. Per capita daily production reached 280.67 g, baseline net inflows were 140.93 g, and the external dependence ratio was only 33.43%. Shanghai’s food-security system is therefore not characterized by generalized scarcity; instead, it exhibits a differentiated structure in which basic energy-supplying foods and nutrient-dense perishable foods are strongly dependent on external corridors, while vegetables retain relatively stronger local support.

**Table 2:** Per capita daily food consumption, local production, net inflows, and external dependence in Shanghai in 2023 (unit: g person^−1^ day^−1^).

Major food group	Subcategory	Per capita daily consumption	Recommended daily intake* (guideline target)	Per capita daily local production	Per capita daily net inflow under normal conditions	External dependence
Grains		462.11	277.78	112.21	349.90	75.72%
Edible vegetable oil		57.36	27.50	0.15	57.22	99.75%
Eggs		64.56	52.17	3.91	60.65	93.94%
Milk (fresh-milk equivalent)		266.90	300.00	33.80	233.11	87.34%
Meat	Pork	132.09	67.23	11.83	120.26	91.04%
Meat	Poultry	87.47		1.58	85.88	98.19%
Meat	Beef	51.63		0.26	65.92	99.60%
Meat	Mutton	14.56				
Aquatic products		163.31	105.82	29.72	133.59	81.80%
Fruit and vegetables	Vegetables	421.60	389.61	280.67	140.93	33.43%
Fruit and vegetables	Fruit	212.56	335.37	31.97	180.59	84.96%

^*^Midpoints of the recommended ranges in the Dietary Guidelines for Chinese Residents (2022) are adopted. The recommended intake for meat refers to total meat intake under the guideline target; meat subcategories are reported separately where consumption, production, and inflow data are available. Effective edible ratios, after accounting for inedible portions and normal losses, are set at 90% for grains, 85% for meats, 89% for eggs, 54% for aquatic products, 82% for fruit, and 77% for vegetables. Beef and mutton are calculated jointly because production and inflow statistics are reported as an aggregate category.

Comparing actual consumption with recommended intake levels further reveals a structural mismatch on the demand side. Actual intake of grains, edible vegetable oil, eggs, meats, aquatic products, and vegetables all exceeded recommended values. Grain consumption, at 462.11 g, was far above the recommended 277.78 g. Edible vegetable oil reached 57.36 g, roughly twice the recommended 27.50 g. Total meat consumption amounted to 285.75 g, greatly exceeding the recommended 67.23 g, while aquatic-product intake of 163.31 g also surpassed the recommended 105.82 g. By contrast, milk and fruit consumption were 266.90 g and 212.56 g, respectively, both below the recommended values of 300.00 g and 335.37 g, with the shortfall in fruit being especially pronounced. Relative to the dietary guideline target, the observed consumption structure in Shanghai can therefore be characterized as high in grains, fats, and animal-source foods but relatively low in milk and fruit. Under the modeled disruption scenarios, maintaining the existing consumption structure would place pressure mainly on the continued external supply of grains, edible oils, and animal-source foods. If the target were shifted toward the dietary guideline structure, milk and fruit would become the key constrained categories in the model. These results describe supply–demand pressure points under specified assumptions, rather than observed household substitution behavior during emergencies. The combined pattern of external dependence and demand-side preference revealed in Table 2 provides the empirical basis for the subsequent identification of static transport thresholds and emergency rationing under sustained disruptions.

### 3.2 Static transport thresholds and bottleneck identification

Based on the static threshold model, and treating total reserves as a system buffer, this study identifies bottleneck categories in Shanghai’s food system and the corresponding minimum transport-capacity ratios under different nutrition-security targets. Table 3 shows that, under the total-stock assumption, dependence on external logistics corridors varies substantially across food categories and that bottlenecks shift markedly when the target changes. Although reserves alleviate immediate supply pressure for some categories, they do not alter the system’s fundamental dependence on external supply networks.

**Table 3:** Static minimum transport-capacity ratios and bottleneck food categories under alternative nutrition-security targets.

Major food group	Subcategory	Reserve stock	Minimum transport-capacity ratio under normal consumption	Minimum transport-capacity ratio under the healthy-diet target
Grains		6931.69	0.00	0.00
Edible vegetable oil		860.40	0.00	0.00
Eggs		19.37	0.68	0.48
Milk (fresh-milk equivalent)		80.07	0.66	0.80
Meat	Pork	39.63	0.67	0.00
Meat	Poultry	26.24	0.69	
Meat	Beef	15.49	0.70	
Meat	Mutton	4.37		
Aquatic products		48.99	0.63	0.20
Fruit and vegetables	Vegetables	126.48	0.10	0.00
Fruit and vegetables	Fruit	63.77	0.65	1.33

Reserve stock is reported in g person^−1^. Transport-capacity ratios are dimensionless. The fruit value of 1.33 under the healthy-diet target is a long-term planning benchmark rather than an operational emergency trigger.

Under the normal-consumption target, the main bottlenecks are concentrated in meat subcategories and other protein-rich or highly perishable foods. Beef and mutton display the highest minimum transport-capacity ratio, at about 0.70, followed by poultry at 0.69 and pork at 0.67. Under the static total-stock assumption, this means that if available external transport capacity falls below roughly 70% of its normal level, meat supply would be the first category to cross the modeled balance boundary. This value should be interpreted as a scenario-based reference point rather than an empirically validated operational trigger. Eggs, milk, aquatic products, and fruit also show relatively high ratios of 0.68, 0.66, 0.63, and 0.65, respectively, indicating that protein-rich and perishable foods are structurally fragile even under normal-consumption conditions. By contrast, the ratios for grains and edible vegetable oil are both zero, and the value for vegetables is only 0.10. Under the static total-stock assumption, local production plus existing reserves are already sufficient to cover most target demand for these categories, leaving their dependence on external logistics relatively limited. The main constraint under the normal-consumption target therefore does not lie in basic energy-supplying foods, but in the rigid external dependence created by high urban demand for animal-source foods and selected perishable foods.

When the target is shifted to the recommended intake levels in the Dietary Guidelines for Chinese Residents, the modeled bottleneck moves from meat to fruit, whose transport-capacity reference value rises to 1.33 under the specified supply, reserve, and demand assumptions. The ratio for milk also increases to 0.80. At the same time, the ratios for grains, edible vegetable oil, vegetables, and meats decline sharply, with grains, edible oil, and vegetables all falling to zero and aggregate meat demand no longer constituting a binding constraint. This result indicates that adopting a more balanced and healthier dietary structure does not automatically reduce pressure on external logistics. Instead, pressure becomes concentrated in nutrient-dense perishable foods such as fruit and milk, which are chronically underconsumed under normal conditions. Shanghai’s food system can still use reserve stocks to provide some buffer when the objective is to maintain observed consumption levels, but if the target shifts toward the dietary guideline structure, stable external supply would remain important for fruit and milk under the model assumptions.

From the perspective of changing demand structure, the relatively high ratios for meat subcategories under the normal-consumption target mainly reflect the fact that current meat consumption far exceeds what local production and reserves can support. The disappearance of meat constraints under the healthy-diet target, by contrast, indicates that once demand is compressed to guideline levels, local supply plus reserves are sufficient to cover recommended intake. Fruit and milk behave in the opposite direction. Their ratios rise rather than fall under the healthy-diet target, revealing a dietary pattern in Shanghai characterized by high meat intake, high fat intake, and relatively low milk and fruit consumption. Total reserve stocks strengthen the short-term buffering capacity of grains, edible vegetable oil, and vegetables, but they are not sufficient to replace the support that external logistics networks provide for nutrient-dense perishable foods. Once the objective shifts from maintaining the observed diet to maintaining a nutritionally balanced diet, system bottlenecks move from traditionally external-dependent animal-source foods to fruit and milk, whose local supply base is weak and whose habitual intake remains inadequate. This finding provides the logical basis for the subsequent analysis of nutrition-constrained emergency standards under sustained disruption, and it suggests that emergency food-security planning in megacities must account for structural differences in bottleneck categories across nutrition-security targets rather than relying exclusively on aggregate reserve expansion.

### 3.3 Minimum energy- and macronutrient-balanced emergency standard under sustained disruptions

When external logistics remain constrained for a sustained period, the model shifts the emergency objective from maintaining the observed consumption structure to ensuring a minimum energy- and macronutrient-balanced emergency standard. To identify the feasible rationing boundary under sustained disruption, the model imposes a minimum energy requirement of 2,100 kcal person-^1^ day^−1^ together with interval constraints for the energy shares of protein, fat, and carbohydrates. Taking effective edible ratios, callable reserves, and the upper bounds of baseline net inflows into account, the linear program solves for the optimal emergency food portfolio under two objectives, namely minimum total external inflow and minimum system bottleneck transport ratio. The results are reported in Table 4. Under the benchmark scenario of H = 15, both objective functions can be satisfied in the model without external inflow, suggesting that the estimated reserve system has potential short-term buffering capacity under the callable-reserve assumption.

**Table 4:** Optimal emergency food ration portfolios under nutrition-constrained minimum-standard scenarios (unit: g person^−1^ day^−1^).

Scenario	Scenario 1: minimum total external inflow, H = 21	Scenario 1: minimum total external inflow, H = 30	Scenario 1: minimum total external inflow, H = 45	Scenario 2: minimum system bottleneck transport ratio, H = 21	Scenario 2: minimum system bottleneck transport ratio, H = 30	Scenario 2: minimum system bottleneck transport ratio, H = 45
Grains	570.90	571.90	572.67	561.80	559.43	559.76
Edible oil	40.02	40.27	40.47	37.98	37.33	37.33
Eggs	4.83	4.56	4.34	12.69	3.91	3.91
Milk	37.61	36.47	35.58	117.23	162.15	161.74
Meats	17.76	16.54	15.58	13.68	13.68	13.68
Aquatic products	32.05	31.35	30.81	29.72	29.72	29.72
Vegetables	286.69	284.89	283.48	286.69	284.89	283.48
Fruit	35.01	34.09	33.39	35.01	34.09	33.39
Protein	77.25	77.03	76.87	78.75	78.75	78.75
Fat	70.00	70.00	70.00	70.00	70.00	70.00
Carbohydrates	302.75	302.98	303.15	301.04	300.97	300.97

H denotes disruption duration in days. The H = 15 scenario is not displayed because both optimization objectives can be satisfied without external inflow. Food-category rows and macronutrient rows are reported in g person^−1^ day^−1^.

Under the objective of minimizing total external inflow, the optimal solution exhibits a clear preference for foods with higher energy density. As disruption duration lengthens from 21 to 45 days, per capita daily grain rations increase modestly from 570.90 g to 572.67 g, while edible vegetable oil rises from 40.02 g to 40.47 g. This model result shows that, under the minimum-inflow objective and the specified nutrient-density parameters, the optimized solution tends to rely more heavily on a grain-oil combination to maintain basic energy supply. By contrast, rations of eggs, milk, meats, and aquatic products decline gradually. Milk falls from 37.61 g to 35.58 g, and meats decline from 17.76 g to 15.58 g, suggesting that under the minimum-inflow objective the system compresses configurations involving lower energy density, stronger substitutability, or relatively higher transport cost. In terms of nutrient mass, protein supply across the three duration scenarios equals 77.25 g, 77.03 g, and 76.87 g, respectively, fat supply remains fixed at 70.00 g, and carbohydrates increase slightly from 302.75 g to 303.15 g. Thus, under the minimum-inflow objective, the model consistently pushes fat supply to the upper bound of the admissible range and mainly compensates for prolonged disruption by increasing the carbohydrate share.

Under the objective of minimizing the system bottleneck transport ratio, the optimal solutions display much stronger multi-category load sharing. Relative to Scenario 1, ration levels for grains and edible oil are slightly lower, while the participation of eggs and milk is substantially higher. Under the H=21 scenario, egg and milk rations reach 12.69 g and 117.23 g, far above the corresponding values of 4.83 g and 37.61 g under Scenario 1. Under H=30 and H=45 , milk stabilizes above 160 g. This indicates that when the objective shifts from minimizing physical inflow volume to minimizing the most stressed transport corridor, external inflows are no longer concentrated in a single major energy-supplying category. Instead, the system enlarges the role of eggs and milk and uses their complementary contributions to protein and fat supply to disperse transport pressure across multiple channels. In nutrient terms, protein supply remains stable at 78.75 g across all three scenarios, fat remains at 70.00 g, and carbohydrates equal 301.04 g, 300.97 g, and 300.97 g, respectively. Compared with Scenario 1, Scenario 2 therefore maintains the same total energy but stabilizes protein supply closer to its upper admissible bound, resulting in a more balanced yet more transport-channel-dependent food configuration.

Taken together, the two optimal solutions define two distinct lower-bound conditions for urban food security under sustained disruption. Scenario 1 identifies the theoretical lower bound of physical external inflow required to maintain the minimum energy- and macronutrient-balanced emergency standard, emphasizing grain- and oil-prioritized support under extreme scarcity. Scenario 2 identifies the minimum transport-capacity condition needed to prevent overload in key logistics corridors, emphasizing wider participation of eggs, milk, and related substitutes to reduce peak pressure on any single corridor. Although the two solutions differ in composition, both satisfy the total-energy constraint and the macronutrient constraints. In all cases, fat converges to the upper bound of 70.00 g, indicating that the fat constraint is binding within the current emergency ration model. This outcome should be interpreted as a consequence of the objective functions, food-category aggregation, and nutrient-density parameters rather than as a behavioral prediction. Because edible oil is highly energy dense and transport efficient, the minimum-inflow objective tends to use oil to satisfy energy requirements with low physical volume, thereby pushing fat intake toward the upper admissible bound. This result highlights the trade-off between transport efficiency and macronutrient balance under the chosen model specification. It also reflects the limitations of using broad food categories, because aquatic products, vegetables, fruit, and meat subcategories contain substantial within-category variation in nutrient density, storage properties, and logistics requirements. Food-security capacity in megacities under sustained disruption therefore depends not only on the total amount of available transport capacity, but also on the minimum availability of critical logistics corridors and the substitutability of food categories in nutrient-equivalent terms.

### 3.4 Sensitivity analysis

To evaluate the robustness of the duration-specific transport-capacity thresholds, we conducted sensitivity analyses by varying three influential assumptions: reserve duration, the benchmark exclusion of fruit and vegetable inflows, and the minimum energy requirement used in the nutrition constraints. The baseline dynamic bottleneck thresholds were 0.34, 0.62, and 0.84 for 21-, 30-, and 45-day disruptions, respectively. When reserve coverage was reduced to 10 days for grains and edible oil and 2 days for perishable foods, the thresholds increased to 0.65, 0.84, and 0.99, indicating that reserve assumptions materially shape the resilience boundary. Conversely, increasing reserves to 20 days for grains and edible oil and 4 days for perishable foods reduced the thresholds to 0.04, 0.40, and 0.69. Allowing fruit and vegetable inflows equal to 10–30% of their normal net inflows lowered the thresholds only modestly; under the 30% allowance scenario, the thresholds declined to 0.33, 0.59, and 0.79. Finally, changing the minimum energy requirement from 2,000 to 2,200 kcal shifted the 21-day threshold from 0.26 to 0.42, the 30-day threshold from 0.54 to 0.70, and the 45-day threshold from 0.76 to 0.92. These results confirm that the ranking of disruption-duration thresholds is robust, but the exact values should be interpreted as scenario-based planning references rather than fixed operational breakpoints (Table 5).

**Table 5:** Sensitivity analysis of dynamic bottleneck transport-capacity thresholds.

Sensitivity setting	H = 21	H = 30	H = 45
Reserve duration			
Low reserves: grain/oil 10 days; perishables 2 days	0.65	0.84	0.99
Baseline: grain/oil 15 days; perishables 3 days	0.34	0.62	0.84
High reserves: grain/oil 20 days; perishables 4 days	0.04	0.40	0.69
Fruit and vegetable inflow allowance			
Fruit/vegetable inflow allowed at 0% of normal net inflow	0.34	0.62	0.84
Fruit/vegetable inflow allowed at 10% of normal net inflow	0.34	0.61	0.82
Fruit/vegetable inflow allowed at 20% of normal net inflow	0.33	0.59	0.81
Fruit/vegetable inflow allowed at 30% of normal net inflow	0.33	0.59	0.79
Minimum energy requirement			
2,000 kcal person-1 day-1	0.26	0.54	0.76
2,100 kcal person-1 day-1	0.34	0.62	0.84
2,200 kcal person-1 day-1	0.42	0.70	0.92

Values denote the minimum system bottleneck transport-capacity ratio under the nutrition-constrained emergency ration model. The baseline assumes 15 days of reserve coverage for grains and edible oil, 3 days of reserve coverage for perishable foods for 10% of the population, zero benchmark external inflow for fruit and vegetables, and a 2,100 kcal person^-1^ day^-1^ minimum energy requirement. H denotes disruption duration in days.

### 3.5 Scenario-based planning thresholds and response priorities

**Figure 1:**
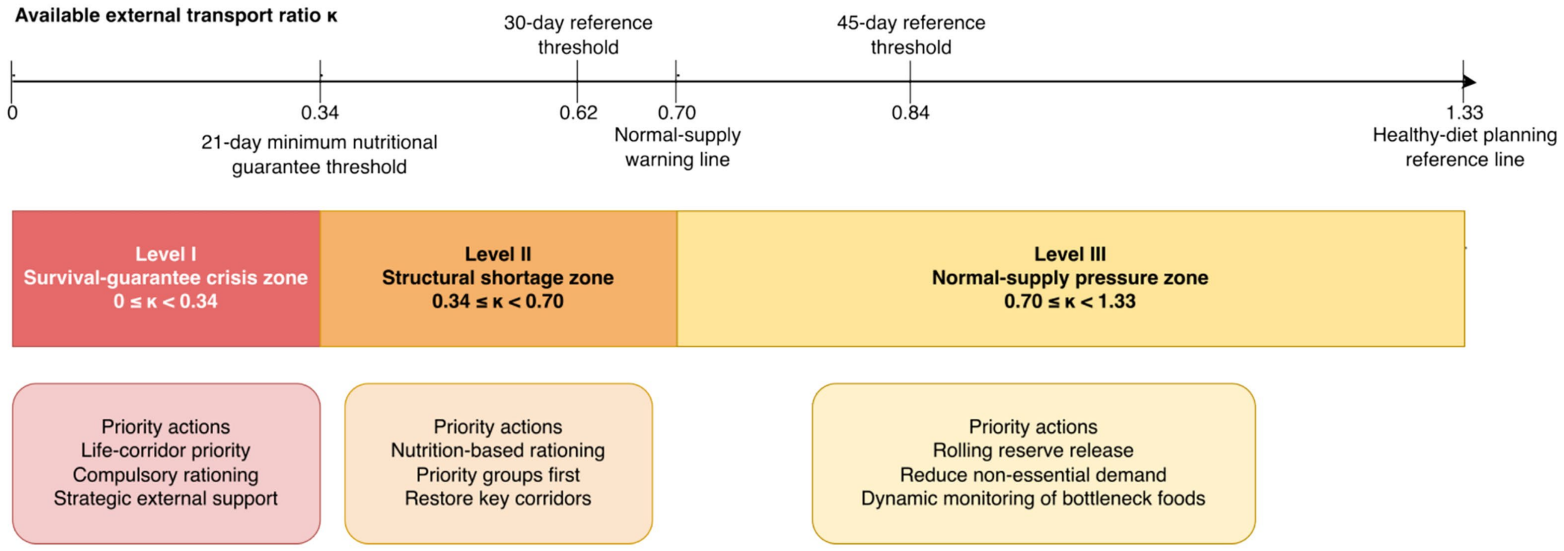
Scenario-based reference thresholds for nutrition-constrained emergency food-security planning.

Based on Tables 3, 4, this study proposes a scenario-based planning framework rather than an operationally validated emergency-warning system. In Figure 1, the value of 0.70 represents a scenario-based reference line for maintaining the observed consumption structure. Under the benchmark assumptions, when the available share of external transport capacity falls below 70% of its normal level, full-category supply under the observed consumption structure becomes infeasible in the model. The value of 0.34 represents the 21-day model-derived boundary for the minimum energy- and macronutrient-balanced emergency standard. Under the benchmark assumptions, when available transport capacity falls below this level, the modeled minimum standard becomes infeasible for the 21-day disruption scenario. The values of 0.62 and 0.84 correspond to the 30-day and 45-day reference points, respectively, and describe the additional transport recovery requirements implied by longer disruptions in the model. The value of 1.33 corresponds to the healthy-diet planning reference line, suggesting that guideline-consistent fruit supply would require additional supply-side or logistics support beyond the current normal benchmark under the specified assumptions. This value therefore serves as a long-term planning benchmark rather than as an operational emergency trigger.

Within this framework, when the available external transport ratio lies between 0.70 and 1.33, the scenario corresponds to Level III, defined here as a pressure zone for normal food-security planning. Supply can still be maintained in broad terms, but the risk of prolonged disruption is already evident. Once the ratio drops below 0.84, the system may still sustain ordinary supply in the short run, yet it can no longer support nutritionally balanced protection under a 45-day disruption. Possible planning priorities in this range include rolling reserve releases, moderation of non-essential consumption, closer monitoring of bottleneck categories such as meat, fruit, and milk, and preparation of interregional dispatch plans. When the ratio falls to the range between 0.34 and 0.70, the scenario corresponds to Level II, defined here as a structural-shortage zone. Under the benchmark assumptions, the observed-consumption target becomes infeasible in the model, and planning priorities would need to shift from maintaining the existing consumption structure to maintaining a minimum energy- and macronutrient-balanced emergency standard. Grains and edible oils become priority categories, while eggs, milk, and aquatic products are rationed in accordance with nutritional substitution, accompanied by targeted protection for priority population groups and simultaneous restoration of key logistics corridors. Once the available transport ratio falls below 0.34, the scenario corresponds to Level I, defined here as a crisis zone for the minimum energy- and macronutrient-balanced emergency standard. Even on a 21-day horizon, the modeled minimum standard is no longer feasible under the benchmark assumptions. Emergency response must then fully shift to lifeline corridor prioritization, emergency rationing, and interregional strategic support. The 15-day reserve-based safety margin suggests that local production and estimated reserves may provide limited buffering under very short shocks, but this modeled capacity is contingent on the callable-reserve assumption and cannot substitute for the recovery of external logistics lifelines.

## 4 Discussion

### 4.1 Principal findings

This study develops a scenario-based, nutrition-constrained framework for identifying emergency food-supply reference thresholds in Shanghai under extreme logistics disruptions. By integrating supply–demand balance, reserve release, external transport capacity, and macronutrient constraints, the analysis suggests that Shanghai’s food-system resilience, under the specified model assumptions, is shaped by the interaction of dietary structure, bottleneck food categories, reserve accessibility, and external logistics availability. These findings should be read as model-based evidence on relative vulnerability rather than as direct forecasts of emergency consumption. The observed divergence between current intake and dietary guidelines identifies potential pressure points, but actual emergency outcomes would also depend on household substitution behavior, affordability, retail access, public procurement, and targeted distribution policies. Three findings are especially important.

First, Shanghai’s food system exhibits high external dependence and a clear mismatch between observed consumption and healthy-diet targets. Edible vegetable oil, beef and mutton, poultry, eggs, milk, aquatic products, and fruit depend heavily on extra-local inflows, whereas vegetables retain a degree of local support. This supply structure overlaps with a dietary pattern characterized by high meat and fat intake and comparatively low milk and fruit intake. As a result, the model identifies highly external-dependent, nutrient-dense, and cold-chain-sensitive categories as potential pressure points under disruption scenarios. Broader cascading effects across the food system are conceptually plausible but are not explicitly simulated in the present framework.

Second, system bottlenecks differ substantially across nutrition-security targets. Under the static total-stock assumption, the main bottlenecks under the normal-consumption target are concentrated in meat subcategories, whereas the principal bottlenecks under the healthy-diet target shift to fruit and milk. Reserve stocks strengthen short-term buffering capacity for basic categories, but cannot substitute for external logistics networks in supporting nutrient-dense perishable foods. Sustained-disruption simulations further indicate that reserve buffers are meaningful mainly for short shocks, whereas longer disruptions rapidly increase dependence on external logistics recovery. Nutrient-equivalent substitution can compress rigid transport demand and extend the resilience boundary, but it does not eliminate the structural role of external logistics lifelines.

Third, static minimum transport-capacity ratios and dynamic response thresholds provide a quantitative basis for scenario comparison and emergency preparedness planning. The static ratio under the normal-consumption target defines the boundary for sustaining full-category observed supply, while the dynamic threshold under the 21-day disruption scenario defines the boundary for a minimum energy- and macronutrient-balanced emergency standard. In the proposed planning framework, different ranges of available external transport capacity correspond to different analytical stress levels. These levels should be understood as model-derived planning categories rather than validated administrative stages. They help indicate when emergency planning may need to move from maintaining observed consumption toward protecting a minimum energy- and macronutrient-balanced emergency standard.

The sensitivity analysis further shows that these reference thresholds are not invariant constants. They are most responsive to reserve-duration assumptions and the minimum energy requirement, whereas partial relaxation of the fruit and vegetable inflow assumption has a smaller but directionally consistent effect. Therefore, the values 0.34, 0.62, and 0.84 should be understood as benchmark results under explicit assumptions rather than empirically validated universal thresholds.

### 4.2 Implications for megacity nutrition-security governance

The Shanghai case suggests that food-security capacity in import-dependent megacities is not determined solely by total food volume or nominal reserve size. It is jointly shaped by the interaction of supply structure, nutritional structure, and logistics structure. Shanghai has built a relatively strong overall food-security base, yet high external dependence, insufficient local provision of nutrient-dense perishable foods, and the upward shift of transport-capacity boundaries under sustained disruption remain salient vulnerabilities. Bottlenecks are concentrated in meat subcategories under the normal-consumption target, but shift to fruit and milk under the healthy-diet target. Although the minimum energy- and macronutrient-balanced emergency standard can be maintained temporarily through local production and reserves, dependence on external logistics lifelines re-emerges as disruptions lengthen. These results point to three broader issues that merit further discussion under the Greater Food Approach.

The Greater Food Approach calls for a broader understanding of food security in megacities, one that avoids reducing staple and non-staple food security to a problem of simple quantity balance. Conventional analyses of urban food security often emphasize whether aggregate supply is sufficient, whether reserve size meets policy requirements, and whether markets can remain stable in the short term. The present results suggest that an important determinant of resilience in the modeled scenarios under extreme disruptions is not aggregate food volume per se, but whether the categories that become bottlenecks under different nutrition-security targets can be supported in a stable manner (39). The marked shift in bottlenecks from meat subcategories under the normal-consumption target to fruit and milk under the healthy-diet target shows that food security in megacities can no longer be understood merely as ensuring that people have enough to eat. It also concerns whether people can continue to eat in a stable, nutritionally appropriate, and sustainable way. The value of the Greater Food Approach for megacity food-security research therefore lies not only in broadening food sources and substitution space, but also in pushing the analytical focus from quantity security alone toward a combined concern with quantity, structure, nutrition, and resilience.

The evaluation standard for megacity staple and non-staple food security should move beyond single indicators such as local self-sufficiency or reserve size. Shanghai’s grains, edible vegetable oil, meat subcategories, milk, aquatic products, and fruit differ sharply in supply origin, nutritional function, and logistics attributes. Under the model assumptions, bottlenecks vary across food-security targets, and transport-capacity reference values shift as disruption duration changes. If food security is assessed only through local self-sufficiency, reserve size, or a single supply–demand gap, the role of highly external-dependent categories, nutritionally important foods, and critical logistics corridors may be underestimated. A more appropriate evaluation framework should integrate supply–demand balance, nutritional structure, reserve capacity, and transport-capacity thresholds, thereby allowing analysts to identify how bottleneck categories evolve under different nutrition-security targets (40). For Shanghai and comparable import-dependent megacities, a more operationally useful question is not whether food security exists in an undifferentiated sense, but under what food-security target, under what disruption duration, and at what level of external transport availability the model begins to reveal structural vulnerabilities.

For Shanghai and comparable import-dependent megacities, emergency food-security planning would benefit from simultaneous attention to reserve coordination, nutritional substitution, and regional linkage, with a gradual shift from static reserve management toward dynamic resilience management. The present results suggest that reserves may play an important cushioning role under short-cycle shocks, yet they cannot fully replace the structural role of external logistics corridors under medium- and long-duration disruptions, especially when reserve mobilization is subject to operational frictions. Nutritional substitution cannot remove that dependence either, but it can reduce rigid external-inflow requirements and lower the peak load on key corridors through cross-category coordination. This means that food-security capacity in megacities should be evaluated not only in terms of total reserves, but also in terms of reserve structure, release efficiency, substitution potential, and interregional coordination. For Shanghai, this implies the need not only to strengthen reserves and deployment mechanisms for grains, oils, and selected bottleneck supplementary foods, but also to deepen emergency coordination across the Yangtze River Delta so as to improve continuity of supply for vegetables, fruit, eggs, milk, and aquatic products. In the longer run, a compound food-security mechanism jointly supported by local supply, reserve buffers, nutrient substitution, and regional linkage is likely to provide a more realistic pathway toward resilient food security in megacities (41).

From a food-policy perspective, the results suggest that emergency food-security planning should not be evaluated only by reserve size or aggregate supply volume. Policy instruments should also consider the nutritional function of food categories, the cost of maintaining buffer capacity for nutrient-dense perishable foods, reserve-release accessibility, and targeted protection for vulnerable groups during prolonged logistics disruptions.

### 4.3 Limitations and future research

Several limitations should be considered when interpreting the results. First, the empirical parameters are calibrated to Shanghai in 2023, and application to other megacities requires re-estimating population, consumption, production, reserve, and interregional supply parameters. The analytical framework may be transferable, but the numerical thresholds should not be directly generalized beyond the Shanghai case. Second, some reserve assumptions rely on policy standards and benchmark cities rather than directly observed stock data or emergency deployment records. The model converts estimated stocks into average daily callable quantities and does not explicitly simulate reserve quality degradation, spoilage, cold-chain interruptions, uneven spatial accessibility, or institutional coordination delays; the estimated reserve-buffering capacity should therefore be interpreted as an upper-bound analytical benchmark rather than as a direct measure of operational deployability. Third, external transport capacity is represented as category-level availability ratios rather than corridor-level network capacity. The model does not explicitly simulate transport-network topology, cold-chain failures, price dynamics, decentralized retail resilience, informal provisioning, or behavioral demand adaptation during emergencies. Fourth, the framework uses average per capita demand and evaluates a minimum energy- and macronutrient-balanced emergency standard rather than comprehensive nutrition security. Micronutrients, dietary diversity, food acceptability, cultural suitability, and vulnerable-population requirements are not explicitly included. Future research could integrate transport-network simulation, interregional supply availability, household-level demand responses, socio-economic heterogeneity, and richer nutritional constraints.

The model treats Shanghai’s population as homogeneous and uses average per capita demand. This abstraction is necessary for system-level threshold estimation, but it does not capture socio-economic inequality in emergency food access. In practice, elderly residents, low-income households, people with limited mobility, and households with special dietary needs may face higher nutrition-security risks even when aggregate supply appears sufficient. Future extensions should integrate vulnerable-group demand, household-level access constraints, and decentralized retail or community distribution capacity.

## Data Availability

The raw data supporting the conclusions of this article will be made available by the authors, without undue reservation.
